# On ‘lost’ indigenous etymological origins with the specific case of the name *Ameiva*

**DOI:** 10.3897/zookeys.748.21436

**Published:** 2018-04-05

**Authors:** Nicole Frances Angeli

**Affiliations:** 1 Division of Amphibians and Reptiles, National Museum of Natural History, Smithsonian Institution, P.O. Box 37012, NHB MRC 162, Washington, DC 20013, USA

**Keywords:** Etymology, indigenous languages, Neotropics, reptiles, seventeenth century, zoological nomenclature

## Abstract

Modern biology builds upon the historic exploration of the natural world. Recognizing the origin of a species’ name is one path to honor the historic exploration and description of the natural world and the indigenous peoples that lived closely with organisms prior to their description. While digitization of historic papers catalogued in databases such as the Biodiversity Heritage Library (BHL) allows for searching of the first use and origin of names, the rapid pace of taxonomic publishing can occlude a thorough search for etymologies. The etymological origin of the genus name *Ameiva* is one such case; while unattributed in multiple recent works, it is of Tupí language origin. The first description was in the *Historiae Rerum Naturalium Brasiliae* by George Marcgrave (1648). *Ameiva* was the name used by Marcgrave’s Amerindian hosts in 17^th^ century Dutch Brazil, where local people spoke the now extinct language Tupí. The Tupí origin was not lost, however, until as recently as the 2000s. Herein, the pre- and post-Linnaean use of the name *Ameiva* is traced and when the name is attributed to the Tupí language and to Marcgrave through time it is noted. The opportunity to discover and/or recover etymological origins, especially names from extinct and indigenous languages, provides insight into the early Western sciences. Careful study of etymology by naturalists is consistent with the idea that science is an evolving process with many predecessors to appreciate.

## Introduction

Our understanding of the relationships of species is evolving rapidly. As a result, scientists are continually revising circumscriptions, proposing new names, and resurrecting old names. Scientific names derived from indigenous, Latin, and Greek words, technical terms, and given in honor of people and places are attributed when known (e.g., Jaeger 1959). The use of indigenous names is frequent, honoring local peoples and places. Best practices associated with indigenous and traditional languages include consultation with native speakers for new attributions or changes to taxa names (e.g., Maori: Whaanga et al. 2013). Sometimes, indigenous words are ‘Latinized’ using the Roman alphabet or with changed endings to follow nomenclatural rules (ICZN Article 11.2-3). In this case, preventative loss of the meaning of the name would ideally involve careful notation of etymological origins. Tracing the early roots of indigenous names given in the past is one way to record historic scientific efforts, honor cultural exchange between Western scientists and the world (Agrawal 1995), and correct mistranslations.

Seventeenth century European naturalists described the fauna and flora of the world widely. Their work is echoed across a multitude of names in use today, common and scientific, derived from indigenous languages. The Sydney language word ‘waratah’ is the common name for the national flower of Australian state New South Wales *Telopea speciossissima* (Sm.) R.Br., while the manatee *Trichechus manatus* Linnaeus, 1758 is a cognate of the Caribbean Taino language ‘manati’. The South American tegu lizard *Tupinambis teguixin* Linnaeus, 1758 is a direct cognate from the extinct language Tupí. The language was spoken widely among Tupinambá people and become the língua geral or the most common unifying Tupí language of the 50 or more languages spoken amongst Tupi-Guarani speaking peoples (Walker et al. 2012).

One of these suspected cognates is *Ameiva* (Meyer, 1795), the modern generic name of a group of more than 36 lizard species distributed throughout Central and South America and the Caribbean. The specific etymology of the name *Ameiva* is marked as ‘unknown’ in some modern taxonomic revisions (Harvey et al. 2012), scientific dictionaries (Beolens 2011), and online databases (Uetz 2015)). If *Ameiva* was Amerindian in origin, usage in natural history literature could help to discover its etymological origins. A digital online resource, the Biodiversity Heritage Library (BHL), allowed a precursory universal search for its use in historic Western science texts and facilitated a starting point. The taxonomic record of the name *Ameiva* was traced to and after Linnaeus, and other scholarship was traced to determine the origin of the name *Ameiva*.

## The *Historiae Rerum Naturalium Brasiliae* (Marcgrave, 1648)

The earliest use of the name *Ameiva* within the BHL was found in the Latin-language *Historiae Rerum Naturalium Brasiliae* by George Marcgrave of the Dutch Republic (Marcgrave 1648). Texts from ancient languages can be difficult to assess, therefore a translation of the original 17^th^ century Latin text is provided (Table 1, Fig. 2). Lizards named *Ameiva* were described in the *Historiae Rerum Naturalium Brasiliae* in 1648 by a brief paragraph, describing the morphology and some behaviors (Marcgrave 1648: 238; Fig. 2).

**Table 1.** Latin to English translation related to the genus *Ameiva* from Marcgrave (1648, p. 238). To view the text as set in the original publication see Figure 2.

**p. 238**	**p. 238**
*Ameiva* Brafiiienfibus & Tupinambis; alia species Lacertorum & superius descriptae Taraguirae per omnia fimilis, excepto quod caudam furcatam habeat; id eft,definentem in duo cornua rexta.	*Ameiva* Brasiiiensibus & Tupinambis; everything is like the other species of lizards described in the group of Taraguirae, except that it has a bifurcated tail. The two tail lengths are straight but vary in measurement.

**Figure 1.**
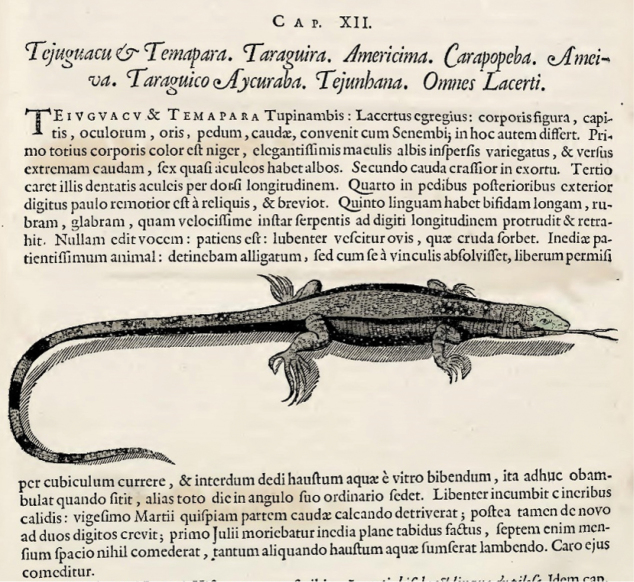
Marcgrave’s watercolors are highly accurate. The watercolor related to the tegu lizard, *Tupinambis teguixin*, is reproduced here set within text (Marcgrave 1648, p. 237).

The first Western use of *Ameiva* in *Historiae Rerum Naturalium Brasiliae* is largely corroborated by taxonomic scholars, both before and after the 1758 publication of Linnaeus’ momentous taxonomic work *Systema Naturae*. Marcgrave wrote and illustrated *Historiae Rerum Naturalium Brasiliae* as an eight-volume book describing the plants, fishes, amphibians, reptiles, birds, mammals, and people of Brazil over a journey beginning in 1638 with Prince John Maurice of Nassau-Siegen (Gudger 1914). Marcgrave accompanied his notes with watercolor paintings made from life in the field over six years and six months (Gudger 1912). Marcgrave returned the final manuscript on a ship bound for Amsterdam but contracted a fever in 1643, and succumbed to death in 1644 without seeing its publication. Jan de Laet, Prefect of the Dutch West India Company, published Marcgrave’s manuscript posthumously (Gudger 1912). The 303-page Latin document contains 429 plates based on Marcgrave’s original watercolor paintings (e.g., see Fig. 1). A later twelve-volume anthology included an additional four chapters related to medical cures of the Americas and become the most popular version of the text (Piso 1658).

The information related to *Ameiva* is accurate based on scientific knowledge of the genus today with the exception ofa description of a bifurcated tail as a diagnostic character. In fact, tail regeneration is quite common across many species and families of lizards and may present as a bi- or trifucation (Bateman and Fleming 2009). Marcgrave’s descriptions of many taxa are highly accurate to this day, specifically depictions of the morphology of fishes and plants (Gudger 1916). The descriptions are referenced by Linnaeus and in pre- and post-Linnaean travel and scientific writings today due to their accurate details (Whitehead 1979).

## Taxonomic nomenclature of *Ameiva* by Linnaeus (1758)

**Figure 2.**
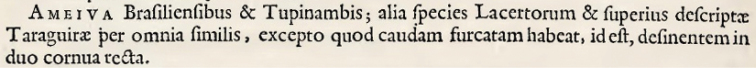
The passage related to the species *Ameiva* that is treated in Marcgrave (1648, p. 238). The Latin text is translated side-by-side with the English in Table 1.

Linnaeus described *Lacerta (= Ameiva) ameiva* in *Systema Naturae* (1758: 203), and cited Seba (1734) and two student dissertations from *Ameoenitates Academicae I* (Linnaeus 1749: 127, 293) in the species description (Bauer 2012; Liner 2012). In Seba’s *Thesaurus rerum naturalium* (Seba, 1734), Seba referenced *Ameiva* from a work of Johannes Jonston (1650:140, table 88, Fig. 2). Barthold Rudolph Hast’s student thesis ‘*Amphibia Gyllenborgiana*’ indicated that Seba described *Ameiva* (1734: 127). Later, Lars Balk (1746) defended a dissertation describing the collections in the ‘*Museum Adolpho-Fridericianum*’ and referenced Hast’s thesis to describe *Ameiva*. Jonston (1650), precursor of Seba, would be a contemporary of Marcgrave. Pre-Linnaean scholarship attributing the description of *Ameiva* to Marcgrave (1648) also exists.

## Historic pre-Linnaean scholarship using *Ameiva*

Outside of Amsterdam, naturalists cited the *Historiae Rerum Naturalium Brasiliae* for many years after its publication (Jonston 1650; Piso 1658; Ray 1686). Johannes Jonston, of the Leszno Academy in Poland, wrote a four volume work, the *Historiae naturalis de quadrupedibus libri* or the ‘Natural History of the Four-footed Beasts’ in 1650, just two years after publication of Marcgrave (Bauer 2012). He described *Ameiva* more than a dozen times across the four volumes. While his contemporaries Piso (1658) and Ray (1686) referenced and quoted various parts of Marcgrave’s work in their natural history writing, Jonston (1650) went so far as to say that Marcgrave was the first to describe American *lacerti* ‘lizards’. Thus, Seba (1734) described *Ameiva* for Linnaeus *sensu* Jonston (1650) and ultimately Marcgrave (1648).

## Post-Linnaean scholarship using the name *Ameiva*

Post-Linnaean publications (Spix 1824; Stejneger 1904) and travelogues (Wied 1825: 86) attributed *Ameiva* to Marcgrave, too. Notably, the German scholar Spix described the ‘brilliantly colored’ *Ameiva* lizards referenced by Marcgrave during the 1817 Bavarian Wied-Neuwied expedition to Brazil (Spix 1824: p. 245). More recently, Stejneger (1904) attributed the name *Ameiva* to Marcgrave in *The Herpetology of Porto Rico* (p. 612). Jaeger (1955) described the word as a root, writing ‘*Ameiv*— name of a kind of lizard’ and Aboriginal in origin. Last, Gotch (1986) recognized both *teju* and *ameiva* as Tupí words.

The species name became generic when Meyer (1795) elevated *Ameiva* from within *Lacerta* Linnaeus, 1758 to a genus with fourteen species of lizards. Meyer (1795) diagnosed *Ameiva* as lizards with five regular parietal scales, prefrontal scales separated from nasal scales, homogeneous lamellae of the toes, without preanal spurs. Meyer (1795) spelled the genus name *Ameiua*, likely because letters like “u” and “j” do not occur in the classical Latin alphabet. Throughout historic scholarship, *Ameiva* reverted to its historic spelling. Other spellings were mistakes, therefore ignored and quickly reverted to *Amaiva* (Kuhl, 1820), *Amieva* (Gray, 1840), and *Amiva* (Cope, 1887).

At the same time, the word *Ameiva* was translated to local languages. Merrem (1820) translated species names into German words that made biological sense to local readers. *Ameiva* was assigned to the German *Warnender*, a ‘traveler’. Blasius Merrem studied reptile and amphibian biology, and he had knowledge of the *Ameiva* as actively foraging lizards. The translation to ‘traveler’ provides context for later species naming including *Ameiva exsul*. Cope (1862) gave the Puerto Rican area *Ameiva* the specific name ‘*exsul*’ without providing context in the original description. The Latin word ‘*exsul*’ has multiple meanings including ‘banished person.‘exile’, ‘wanderer’, and ‘traveler’. Stejneger (1904: 612) knew Cope personally and added a footnote to *The Herpetology of Porto Rico* (Stejneger 1904) that the Latin ‘*exsul*’ translates to ‘wanderer or traveler’. At least one later scholar without knowledge of the Stejneger (1904) footnote, translated ‘*exsul*’ as a ‘banished person or exile’, losing the spirit of the original attribution (Brown 1954). Although relatively minor consequences exist in the misunderstanding of the original meaning of *Ameiva*, this represents just one example of the importance ofmaintaining a clear and consistent record of etymologies.

Other authors developed Marcgrave’s work with their own flourishes. Owens (1742), a popular 18^th^ century writer, described *Ameiva* as fearful creatures with two tails, concluding that ”….this Article [*forked tail*] seems to differ from all other sanguineous Animals….I have never heard of any else furnished with two Tails (p. 122)”. As stated earlier, birfucation is common amongst Neotropical lizards. Owens’ (1742) other colorful writing declared the *Tejuguacu* (=*Tupinambis teguixin*) could for “…six or seven moons, live without any sustenance, but air, the fluid in which we all breathe”. Marcgrave did tether a tegu to a leash in his Brazilian residence (Fig. 1). The tegu refused to eat and wasted away to its death after living without sustenance for seven months: “septem enim mensium spacio nihil comederat”.

## Records of extinct languages

Now establishing that Marcgrave ran into the word *Ameiva* in South America, of local Amerindian origin, a problem exists in independently verifying the word by Tupí language authorities. The word *Ameiva* was not found in available Tupí dictionaries and references searched (da Silveira Bueno 1998; Tibirçá 2001; Chiaradia 2015). Henrique Caldeira Costa (pers. comm.) speculates that the name *Ameiva* is actually a contraction of two Tupí words, ‘Ambere’ and ‘Aíba’ meaning ‘lizards that are not fit to eat’. Chiaradia (2015) defines ‘Aimbere’ or ‘Ambere’ as ‘one who writhes’, a reasonable translation for lizards. Tibirçá (2001) translates ‘amberé’ to the Portuguese ‘lagartixa’, a small lizard. Chiaradia (2015) and Tibirçá (2001) translates ‘Aiba’ to ‘something that is not good to eat’ or ‘bad/evil’.What Marcgrave may have heard spoken in 1640 was ‘amberé – aíba’ or writhing, inedible lizards (H. Cladeira Costa, pers. comm.). Only the history of the name *Ameiva* was discussed herein; however, Marcgrave described many more Neotropical species. Other examples in the botanical and zoological literature from the Tupí language groups include *piranha*, *jacaranda*, *petunia*, and *jaguar*.

## Conclusions

An Amerindian origin exists for the word *Ameiva*, possibly old Tupí, first introduced into the Western science vernacular by Marcgrave (1648). Etymology is germane in a world of rapid scientific discourse. Negligible time is required to determine the origin of many scientific names unattributed in modern scholarly works. With the digitization of historic texts in online databases (i.e., Biodiversity Heritage Library), we are linked more easily today to our academic predecessors than any other time in the recent past (Pilsk et al. 2010). Correcting the attribution of the word *Ameiva* honors scientific inquiry of the past and historic contributions of indigenous people to Western scientific nomenclature. The *Historiae Rerum Naturalium Brasiliae* is digitized and searchable and so may provide important insights into the behavior, distribution, and historic assemblage of species in eastern South America (Lees and Pimm 2015). For groups in need of taxonomic and phylogenetic revision, like the family *Teiidae* containing the genus *Ameiva* (Harvey et al. 2012; Pyron et al. 2013), great opportunity exists for recovering lost etymologies and new honorarium.
